# Simultaneous Temperature Measurements and Aerosol Collection During Vaping for the Analysis of Δ^9^-Tetrahydrocannabinol and Vitamin E Acetate Mixtures in Ceramic Coil Style Cartridges

**DOI:** 10.3389/fchem.2021.734793

**Published:** 2021-08-09

**Authors:** John Lynch, Lisa Lorenz, Jana L. Brueggemeyer, Adam Lanzarotta, Travis M. Falconer, Robert A. Wilson

**Affiliations:** Forensic Chemistry Center, Office of Regulatory Science, Office of Regulatory Affairs, U.S. Food and Drug Administration, Cincinnati, OH, United States

**Keywords:** EVALI, vaping, temperature, vitamin E acetate, Δ^9^ -tetrahydrocannabinol, ceramic coil

## Abstract

Incidence of e-cigarette, or vaping, product use-associated lung injury (EVALI) has been linked to the vaping of tetrahydrocannabinol (THC) products to which vitamin E acetate (VEA) has been added. In this work we vaped THC/VEA mixtures at elevated power levels using a variety of ceramic coil vaping cartridges and a commercially available vaping device, while simultaneously measuring temperature and collecting the vaporized condensate. The collected vapor condensate was analyzed for evidence of VEA decomposition by GC/MS, GC/FT-IR/MS, and LC-APCI-HRMS/MS. Mean temperature maxima for all examined cartridges at the selected power exceeded 430°C, with a range of 375–569°C, well beyond that required for thermal decomposition of VEA. The percent recovery of VEA and Δ^9^-THC from the vaporized mixture in six cartridges ranged from 71.5 to 101% and from 56.4 to 88.0%, respectively. Analysis of the condensed vaporized material identified VEA decomposition products duroquinone (DQ), 1-pristene, and durohydroquinone monoacetate (DHQMA); a compound consistent with 4-acetoxy-2,3,5-trimethyl-6-methylene-2,4-cyclohexadienone (ATMMC) was also detected. The concentration of DQ produced from vaporization of the THC/VEA mixture in one cartridge was found to be 4.16 ± 0.07 μg per mg of vapor condensate.

## Introduction

In August of 2019, reports of an increasing number of hospitalizations for respiratory difficulties, and in some cases acute respiratory failure, among users of e-cigarettes and other vaping products started to emerge. The outbreak of e-cigarette, or vaping, product use-associated lung injury (EVALI) peaked in September of 2019. As of February 2020, 2,807 people had been hospitalized across the U.S., including Puerto Rico and the U.S. Virgin Islands, with 64 deaths. (U.S. Centers for Disease Control and Prevention, 2020). Investigations into the cause of EVALI were initiated at state and federal laboratories, including the CDC, and the U.S. Food and Drug Administration (FDA). These efforts mainly focused on whether components of the e-liquids being vaped were responsible for the injuries observed.

According to statistics compiled by the CDC, of the 2,022 patients for whom data were available, 82% (as of January 14^th^, 2020) reported using tetrahydrocannabinol (THC)-containing products, including 33% who claimed to use THC-containing products exclusively. (U.S. Centers for Disease Control and Prevention, 2020). In September of 2019, the New York State Department of Health announced that in an examination of 34 EVALI patients who used THC-containing products, all used at least one product that also contained vitamin E acetate (α-tocopherol acetate, VEA), a compound which was not an approved additive for New York State Medical Marijuana Program-authorized vape products. (Department of Health, 2019). The FDA reported that, in a sample of 93 verified EVALI patients, 73% were linked to at least one THC-containing product, and 81% of those THC-containing products also contained VEA. (U.S. Food and Drug Administration, 2020). The FDA also found that in a broader analysis of 511 THC-containing products, 50% contained VEA as a diluent, with concentrations ranging from 23 to 88%. (U.S. Food and Drug Administration, 2020). In a study of bronchoalveolar-lavage (BAL) fluid from 51 confirmed or probable EVALI patients, 47 (92%) were linked to the use of THC-containing products, and the BAL fluid of 48 (94%) contained VEA. (U.S. Centers for Disease Control and Prevention, 2020). VEA was not found in the BAL fluid of a comparison group of 99 healthy individuals. (U.S. Food and Drug Administration, 2020). Subsequently, an animal study found that mice exposed to an 88% VEA aerosol showed histological markers consistent with EVALI. (Bhat et al., 2020). In addition to VEA, studies of EVALI-associated THC products have revealed the presence of medium-chain triglycerides (also sometimes used as a cutting agent) in some products, as well as residual organic solvents, silica compounds, and pesticides. (Raber et al., 2015; Muthumalage et al., 2020). The CDC has issued guidance to avoid the use of any e-cigarette or vaping product containing VEA, and any product obtained from informal sources. Both the CDC and FDA caution, however, that although there appears to be a strong link between VEA and EVALI, the evidence is insufficient to rule out other contributing factors, and the precise mechanism by which VEA may cause injury is unknown. (U.S. Centers for Disease Control and Prevention, 2020; U.S. Food and Drug Administration, 2020; Blount et al., 2019; Lanzarotta et al., 2020).

While the types of products associated with EVALI have been well characterized, one variable that requires deeper consideration is the impact of vaping temperature on these products. Vaping, whether of nicotine or THC products, can be done over a range of power levels, determined by the resistance of the heating element and the voltage applied by the vaping device. Many devices allow the user to set the voltage or temperature used to vape, with varying accuracy. (Dibaji et al., 2018). Identifying a common or uniform practice in vaping power levels is difficult. However, it has been shown that vaping at higher power levels results in higher temperatures during vapor generation, and differences in temperature can affect many features of the vapor generated, including volume, particle size and particle size distribution, appearance, and flavor. (Varlet et al., 2016; Talih et al., 2017; Soulet et al., 2018).

Differences in temperature, especially at elevated power, may also result in thermal decomposition of one or more components of the e-liquid. (Geiss et al., 2016; Bitzer et al., 2018; Meehan-Atrash et al., 2019). The potential for components of THC and VEA-containing products to be converted to more toxic species at higher temperatures merits further investigation, and may be a factor in the causation of EVALI by vaping mixtures that include VEA. (Blount et al., 2019; Bhat et al., 2020). A thermogravimetric study found that VEA decomposition begins at around 200°C in air, and accelerates rapidly above 300°C. (Ushikusa et al., 1991). Another study catalogued a number of products of the thermal degradation of VEA between 180 and 300°C, including formic acid, acetic acid, and 2-hexanone. (Riordan-Short et al., 2019). Both of these studies examined the decomposition of VEA over temperature exposures lasting several minutes, well in excess of the few seconds of exposure produced during vaping.

An examination of VEA aerosolized by vaping demonstrated the potential for the generation of ketene gas (C_2_H_2_O), a toxic pulmonary irritant (National Research Council, 2014), from thermal decomposition of the aryl acetate moiety of VEA. This study was performed using a device designed for nicotine vaping, producing very high power using a sub-Ohm (0.25 Ω) resistance coil (Wu and O’Shea, 2020), in contrast to the ceramic wick cartridges commonly used for vaping of THC concentrates, with typical coil resistances in the 1.3–2.2 Ω range. One study has identified and quantitated the VEA decomposition products duroquinone and durohydroquinone in a vaporized VEA solution in one 1.4 Ω ceramic wick cartridge operated at 3.6 V (Jiang et al., 2020). Another study on VEA aerosolization monitored the temperature of a cartridge with a stainless steel coil by IR temperature measurement through a custom designed cell following the removal of the tank and found temperatures from 500–600°C, though no temperature data on ceramic coil cartridges was provided. (Mikheev et al., 2020). The operating temperature and the effects of elevated temperature, regarding the thermal decomposition of VEA in ceramic coil cartridges designed for the vaping of THC concentrates have not been well characterized.

In this work, we use a variety of THC concentrate compatible cartridges and a commercially available vaping device with an adjustable voltage setting to monitor the temperature at multiple power levels and examine the effects of sustained vaping at elevated power levels on a mixture of THC concentrate and VEA (THC/VEA). The THC/VEA concentrations and cartridges used were representative of products associated with EVALI. We then collected and compared the chemical composition of the vapor condensate produced with the unvaped mixture to identify any thermal decomposition products created from vaping the THC/VEA mixture.

## Experimental

### Samples

A 50% THC-concentrate, 50% VEA mixture (w/w, THC/VEA) was prepared by combining approximately equal weights of a THC concentrate collected during an investigation into the alleged manufacturing and distribution of illicit vaping cartridges (75% Δ^9^-tetrahydrocannabinol, verified by HPLC-UV, also contained cannabinol, cannabigerol, cannabidiol, and Δ^9^-tetrahydrocannabivarin identified by GC/MS) and commercially available diluting agent (100% VEA, verified by GC-FID). The mixture was then heated to 90°C for 45 min and vortexed. Prior to dispensing into cartridges, the THC/VEA mixture was again heated to 90°C for at least 30 min to decrease viscosity. Between 0.5 and 0.9 g of the mixture was dispensed into empty 510 threaded vaping cartridges identified C1-C6 (various ceramic coil type cartridges are shown in Supplementary Figure S1) using a 3 ml Luer-Lok syringe with a 16G needle (BD Precision Glide Needle, Becton, Dickinson and Company, Franklin Lakes, NJ).

Cartridges C1, C2, C3, C4, and C6 were ceramic coil type cartridges purchased online to represent the types of THC containing cartridges commonly seen during the EVALI investigation and C5 was a ceramic coil type cartridge locally purchased. Additionally, one black market and one authentic (commercially available in a jurisdiction where such products are legal according to state and local law) cartridge containing Δ^9^-tetrahydrocannabinol received by the FCC as part of the EVALI investigation were used. The resistance of each cartridge shown in Supplementary Figure S1 was measured prior to use with a multimeter (FLUKE 79 Series II Multimeter, Fluke Corp., Everett, WA).

An unvaped portion of the THC/VEA mixture was dispensed into a section of tygon tubing (0.19’’ internal diameter (ID), Masterflex L/S 15 E-3606, Cole-Parmer, Vernon Hills, IL, United States), allowed to rest 15 min, and then collected for use as a control.

### Vaping Apparatus for Aerosol Production and Collection

**FIGURE 1:**
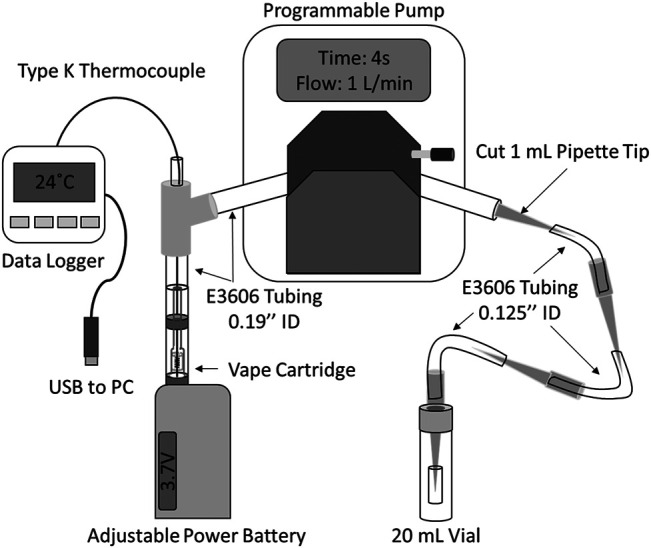
Diagram of vaping apparatus (not to scale) for temperature monitoring and condensed vapor collection.

A direct method for aerosol generation and collection was adapted from previous work (Olmedo et al., 2016; Lanzarotta et al., 2020) to allow for the insertion of a thermocouple for temperature measurements as shown in Figure 1. Aerosol was generated using a peristaltic pump (drive no. 07522-20 and head no. 77200-62, Cole-Parmer, Vernon Hills, IL, United States), which pulls the aerosol from a cartridge attached to a vaping device (Eleaf iStick 30W, locally purchased). The cartridge was connected to a y-junction by a variable length of 0.19’’ ID tygon tubing (approximately 5–8 cm) fit snugly over the cartridge mouthpiece. From the y-junction, a 20 cm piece of 0.19’’ ID tygon tubing was run through the peristaltic pump. Downstream of the pump, the 20 cm tube was connected to a series of four 1 ml pipette tips (one cut to fit inside the 0.19 ID tubing and the rest uncut to fit over the 1/8’’ ID tubing), three 6 cm sections of tygon tubing (E-3606 tygon, 1/8’’ ID tubing, Cole-Parmer, Vernon Hills, IL, United States), and one 2 cm section of 1/8’’ ID tygon tubing. The last pipette tip and 2 cm of tubing was inserted loosely into a hole in the cap of a 20 ml glass vial, to allow for ventilation. The tubing and pipette tips were used as received and all tubing and pipette tips were replaced following vaporization of each cartridge.

For all vaping experiments the peristaltic pump was programmed to operate at a flow rate of 1.0 L/min and a puff topography of 4 s puffs every 30 s, with manual activation of the vaping device starting 3 s prior to puff start at a voltage set according to experimental design. This puff topography is similar to that of an average experienced e-cigarette slow user as described by Talih et al. (2015). (Talih et al., 2017). Condensate was collected by centrifuging (Eppendorf 5810 centrifuge, Eppendorf, Hauppauge, NY) each portion of the tubing system containing condensed material at 4,000 rpm for 2 min into a glass vial following each experiment.

### Measuring Temperature

Temperature was measured during vaping by threading a Type-K thermocouple probe (Models TJ36-CASS-020G-6 and TJ36-CASS-020U-6, Omega Engineering Inc., Norwalk, CT) through a sealed junction in the vaping apparatus tubing and down through the mouthpiece and airway to the coil/wick of each cartridge (Figure 1). Temperature measurements were very sensitive to the position of the probe tip, with the highest temperature measurements obtained when the probe tip was located between the top and bottom of the heating coil. Except where otherwise stated, probes were positioned to read the maximum temperature during vaping. Temperature data was logged at 1 s intervals throughout each experiment with a portable data logger (OM-74, Omega Engineering Inc., Norwalk, CT). The thermocouple probes were cleaned following vaporization of each cartridge by rinsing with methanol (Fisher Scientific, St. Louis, MO, United States) and water (18 MΩ purity, Millipore, Massachusetts, United States) while gently wiping the exterior with Kimwipes to remove any excess material. The probes were replaced if they appeared damaged or the material could not be sufficiently removed.

### GC-FID Analysis

An internal standard solution was prepared by weighing a portion of squalane, purchased from USP (Rockville, MD, United States), into a volumetric flask that yielded a 2.5 mg/ml solution when diluted to volume with cyclohexane, purchased from Fisher Scientific (St. Louis, MO, United States). The solution was sonicated for approximately 5 min. All samples and vitamin E acetate standards were diluted using the internal standard solution. The vitamin E acetate standard was purchased from USP.

Adapting previously developed methods (Ciolino et al., 2018a), triplicate preparations of approximately 10 mg from cartridges C1-C6 and one unvaped portion of sample were combined with 250 µl of internal standard solution. Vials were vortexed to mix and sonicated for approximately 10 min. An additional 1:40 dilution was prepared from the initial dilution. A portion of C6 was spiked with vitamin E acetate at a level 480 mg/g.

The vitamin E acetate stock standard was prepared at approximately 10 mg/ml. A calibration curve was constructed from dilutions of the stock standard. The vitamin E acetate curve was linear 10 µg/ml to 1 mg/ml, with r = 0.9999 for duplicate injections of the high and low standard and five injections of the mid standard. Separation and detection of VEA was conducted using a GC-FID. Chromatography was performed on an Agilent Technologies (Santa Clara, California, United States) GC 7890B Series outfitted with a G4567A Series autosampler and an Agilent HP 5% phenyl column with length, I.D., and film thickness dimensions of 30 m, 0.32 mm, and 0.25 µm, respectively. Helium carrier gas was employed in constant flow mode using a flow rate of 1.3 ml/min. Injections were performed in a 50:1 split mode with an injection volume of 1.0 µl and an injector temperature of 290°C. The method included a starting temperature of 60°C with a hold time of 0.5 min then a ramp rate of 25°C/min until a temperature of 220°C was reached and held for 10.0 min. A second ramp rate was performed of 10°C/min until a final temperature of 300°C was reached and held for 9.0 min, which resulted in a total run time of 33.9 min.

Flame ionization detection was accomplished using an Agilent Technologies FID. A H_2_ flow rate of 30 ml/min, an airflow rate at 400 ml/min, and 25 ml/min helium makeup gas flow were used. The temperature of the detector was set at 300°C. Data were acquired and analyzed using Agilent OpenLab CDS software version C.01.07 SR2 [255].

### HPLC-UV

Adapting previously developed methods (Ciolino et al., 2018b), the material from cartridges C1-C6 and one unvaped portion of sample was heated to 90°C for approximately 5 min to ensure ease of sample transfer. Triplicate preparations of approximately 25 mg of material from each were weighed and placed into 4 ml glass vials. 1.0 ml of 95% ethanol ACS USP Grade (Fisher Scientific, St. Louis, MO, United States) was added to each vial, capped and vortexed. A 0.1 ml aliquot of each initial dilution and 10.0 ml of 95% ethanol were added to a scintillation vial, capped, vortexed and then filtered using 0.45 μm nylon membrane filter. A Δ^9^-THC standard, purchased from Cerilliant (Round Rock, TX, United States), was used to prepare an external calibration curve.

Analysis was performed on an Agilent HPLC system with UV detection (1,260 Infinity) with an ACE 5 C18-AR, 250 × 4.6 mm, 5 μm HPLC column. Column compartment temperature was maintained at 30°C. Using isocratic conditions, the mobile phase consisting of 0.5% acetic acid and acetonitrile (34:66). The total run time was 60 min with a flow rate of 1.0 ml/min, UV detection at 240 nm, spectral collection, 190–400 nm, and injection volume of 25 µl.

### GC/MS Analysis

Adapting previously developed methods (Ciolino et al., 2018a), duplicate preparations of approximately 25 mg from cartridges C1-C6 and one unvaped portion of sample were combined with 1 ml of 95% ethanol. Vials were vortexed to mix. 25 µl of the sample extract was added to 1 ml of acetonitrile and vortexed to mix. 200 µl of sample extract was derivatized using pyridine and BSTFA + 1% TMCS (bis(trimethylsilyl)trifluoroacetamide +1% Trimethylchlorosilane**).**

Separation and detection for the underivatized and derivatized sample preparations were conducted using a GC/MS. Chromatography was performed on an Agilent Technologies GC 7890B Series outfitted with a 7963 Series autosampler and an Agilent 5% phenyl column for the underivatized and a Restek 35% silphenylene column for the derivatized, both with length, I.D., and film thickness dimensions of 30 m, 0.25 mm, and 0.25 µm, respectively. Helium carrier gas was employed in constant flow mode using a flow rate of 0.8 ml/min. Injections were performed in a splitless mode with an injection volume of 1.0 µl and an injector temperature of 250°C. The method included a starting temperature of 60°C with a hold time of 0.5 min then a ramp rate of 25°C/min until a temperature of 220°C was reached and held for 10.0 min. A second ramp rate was performed of 10°C/min until a final temperature of 300°C was reached and held for 15.0 min, which resulted in a total run time of 39.9 min.

Mass spectrometric detection was performed using an Agilent 5977B series mass selective detector. Data were collected with a mass range of 40–600 Da using full scan mode, a 3.5 min solvent delay for the underivatized and a 7.0 min solvent delay for the derivatized, a threshold of 150, quadrupole temperature of 150°C, a source temperature of 230°C, and electron ionization energy of 70 eV. Data analysis was performed using Agilent Mass Hunter Version B.07.06.2704.

### GC/FT-IR/MS Analysis

Sample extracts from the GC/MS preparation were examined. 50 µl of acetonitrile was added to 50 µl of the C5 cartridge sample extract. A duroquinone standard was purchased from Sigma (St. Louis, MO, United States), and prepared and analyzed at a concentration of 0.5 mg/ml.

Separation and detection of the C5 cartridge extract was conducted using a fully integrated GC/FT-IR/MS instrument. Chromatography was conducted using an Agilent 7890B Series GC outfitted with a G4567A Series autosampler and a Phenomenex Zebron ZB-5MSi column with length, I.D. and film thickness dimensions of 30 m, 0.25 mm, and 0.25 μm, respectively. Helium carrier gas was employed in constant flow mode using a flow rate of 2 ml/min. Injections were performed in a splitless mode with an injection volume of 1.0 μl and an injector temperature of 250°C. The method included a starting temperature of 75^o^C with a hold time of 1.0 min and a ramp rate of 30°C/min until a final temperature of 330°C was reached. The final temperature was held for 10.5 min, which resulted in a total run time of 20 min. The terminus of the column was inserted into an inert capillary tee that splits approximately 66% of the GC effluent to a transfer line connected to the IR interface and approximately 34% of the GC effluent to a transfer line connected to the MS interface. The transfer line temperatures from the GC to the mass-selective detector and from the GC to the IR detector were 280 and 300°C, respectively.

Infrared detection was accomplished using a Dani Instruments DiscovIR FT-IR spectrometer. The terminus of one transfer line exiting the inert capillary tee from the GC was inserted into the IR interface and positioned directly above the ZnSe disk. Data were collected using a 100 μm × 100 μm MCT detector, 4,000–700 cm^−1^ spectral range, 4 cm^−1^ resolution, 10 mm/min disk speed, 5.0 min solvent delay, 300^o^C restrictor temperature, 300°C oven temperature, 35°C dewar cap temperature and −40°C disk temperature. Instrument operations and data analysis were conducted using workbooks designed in Grams software version 9.2 by Dani Instruments.

Mass spectrometric detection was performed using an Agilent 5977A series mass selective detector. The terminus of the second transfer line exiting the inert capillary tee from the GC was inserted into the MS and positioned directly in front of the electron ionization (EI) source. Data were collected with a mass range of 50–550 Da using full scan mode, a 5.0 min solvent delay, detector turned off at 8 min, −125 relative voltage, a threshold of 150, quadrupole temperature of 150°C, a source temperature of 230°C, and electron ionization energy of 70 eV. Data analysis was performed using Agilent MSD Chemstation software version F.01.03.2357.

### LC-APCI-HRMS/MS Qualitative and Quantitative Analysis

The samples prepared for GC/MS analysis were further diluted by a factor of 10 in 50/50 H_2_O/MeOH for qualitative analysis. A 42.0 mg portion of sample C1 was dissolved in 1 ml of acetonitrile for quantitative analysis; five 50.0 μl aliquots of this solution were each combined with 950 μl of methanol. Four of these diluted solutions were spiked with appropriate amounts of a 1.00 mg/ml solution of duroquinone reference standard to measure the level of duroquinone by standard additions.

Qualitative analysis was performed on a Thermo Scientific Dionex UltiMate 3000 LC equipped with a Phenomenex Luna Phenyl-Hexyl, 3.0 µm, 2.0 × 150 mm column held at 40°C, coupled to a Thermo Scientific Q Exactive high-resolution mass spectrometer (HRMS) equipped with an atmospheric pressure chemical ionization (APCI) source. Data were acquired using Xcalibur 4.0 software from Thermo Scientific. Mobile phase flowed at a constant rate of 0.250 ml/min. Following a 5-min pre-injection equilibration at 90% A (18.2 MΩ∙cm H_2_O) and 10% B (HPLC-grade MeOH), gradient elution was performed by linearly ramping to 95% B in 20 min and holding for 12 min. Eluant was directed to the HRMS inlet from 2.5 to 21 min and diverted to waste at all other times to avoid saturation of the HRMS with high levels of THC and VEA. The injection volume was 5.0 μl.

The instrument parameters for the mass spectrometer were as follows: positive polarity; sheath gas flow = 25 arbitrary units; auxiliary gas flow = 5 arbitrary units; sweep gas flow = 2 arbitrary units; APCI temperature = 250°C; discharge current = 5.0 μA; capillary temperature = 275°C; S-lens RF level = 60; resolution = 140,000 (full scan), 35,000 (MS/MS); automatic gain control (AGC) target = 1 × 10^6^; scan range for full scan data collection = *m/z* 130–1,200. Data-dependent MS/MS spectra were collected on either the two most abundant ions in the preceding full scan spectrum, or from an inclusion list that included only duroquinone (DQ) and 4-acetoxy-2,3,5-trimethyl-6-methylene-2,4-cyclohexadienone (ATMMC), employing a 1.0 s dynamic exclusion window, ±0.5 Da isolation window, and collision energy of 30 eV. The instrument was calibrated according to manufacturer’s specifications.

Quantitative duroquinone analysis was performed using the same LC, column, mobile phase, and flow rate, but using a 4-min pre-injection equilibration at 40% A and 60% B, followed by isocratic elution for 14 min, followed by a step to 90% B and holding for 10 min. Eluant was directed to the HRMS inlet from 2 to 13.5 min and diverted to waste at all other times. The injection volume was 2.0 μl and each solution was injected in triplicate. The HRMS ion source parameters were identical, but data acquisition was limited to MS/MS spectra of *m/z* 165.0910 ± 0.5 at a collision energy of 30 eV. Extracted ion chromatograms (EICs) of *m/z* 107.0495 (±0.0005) were used for measuring duroquinone peak areas.

## Results and Discussion

### Temperature Measurements and Aerosol Collection

**FIGURE 2:**
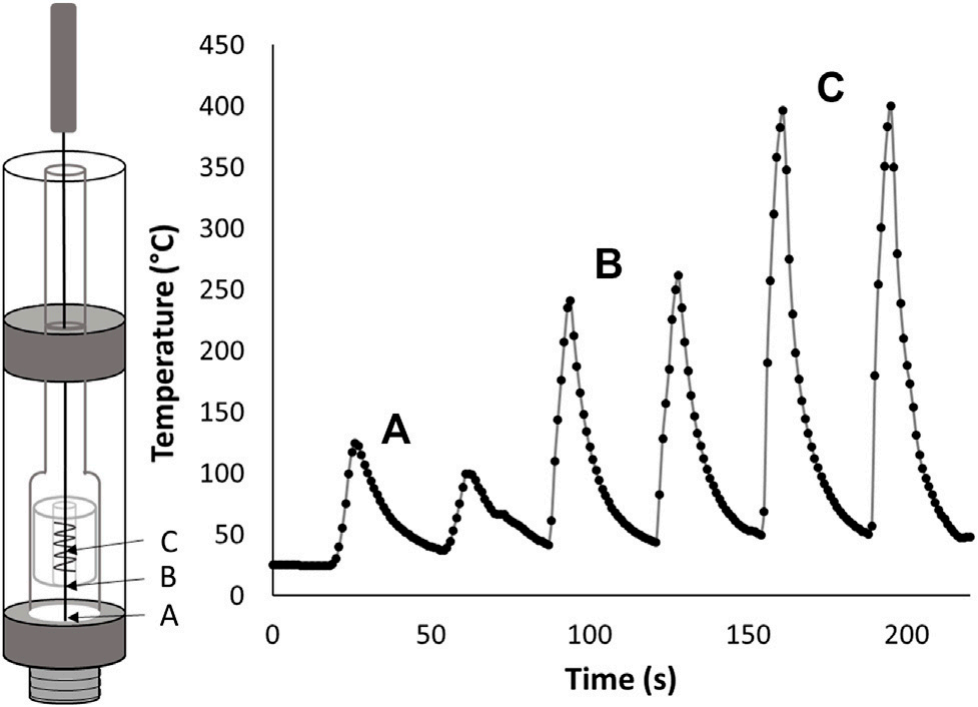
Thermocouple placement inside of a black-market ceramic coil cartridge containing Δ^9^-tetrahydrocannabinol with 1.5 Ω resistance and applied battery voltage of 3.7 V **(A)** Near the bottom of the cartridge. **(B)** Slightly below the bottom of the ceramic coil. **(C)** Inside the ceramic coil near the center.

To allow for temperature measurements that are the most representative of a typical ceramic coil cartridge user experience, the cartridges were left completely intact. While this is advantageous for mimicking the end user experience it makes exact positioning of the probe in the cartridge difficult because the coil cannot be seen from the outside of the cartridge. To examine the effect of probe placement in the cartridge, the temperature was monitored at multiple sites from the bottom of the cartridge to the estimated center of the ceramic coil as shown in Figure 2. For the cartridges tested in this work there was generally approximately 3–5 mm of space between the bottom of the coil and the bottom of the cartridge. The total coil lengths for the cartridges used in this work were approximately 5 mm. This gives an approximate 8–10 mm of space from the bottom of the cartridge to the top of the coil. Based on our experience, these dimensions were similar for all ceramic coil type cartridges.

Figure 2 shows extreme differences in temperature measurements are seen based on probe placement. At or near the bottom of the cartridge, temperatures in the 100°C range were seen while movement to the estimated center of the cartridge only approximately 5.5–7.5 mm away, produced temperatures of 400°C. Moving only about 3 mm away from the center of the coil to at or just below the bottom of the coil results in temperatures of 250°C.

Similar results were seen but not shown for movement of the probe above the coil. The clear temperature difference within the cartridge makes probe positioning an essential part of accurately measuring the maximum temperature. To ensure proper probe placement for all vaping experiments performed in this work, the top of the coil was identified by inserting the probe gently until resistance from the top of the coil was felt then slowly feeding the probe an additional 2.5 mm into the cartridge. During the initial vaping for each cartridge the probe was moved slightly up or down until the placement resulting in the highest temperature was found. The probe was kept at this position for the remainder of the vaping process.

**FIGURE 3:**
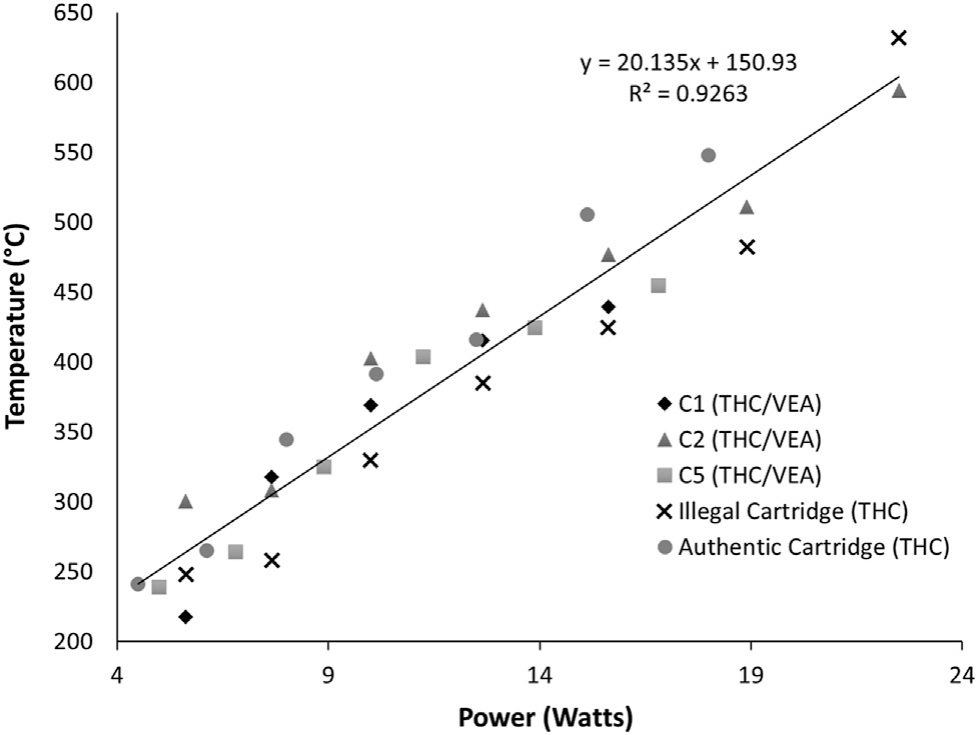
Maximum temperature measured (*n* = 3) for five cartridges at battery voltage settings of 3.0, 3.5, 4.0, 4.5, 5.0, 5.5, and 6.0.

To determine the temperature associated with different voltage settings and the maximum power level at which the THC/VEA mixture could be sustainably vaped, we examined the temperature response of five different cartridges during vaping at increasingly higher power levels (Figure 3). Three cartridges (C1, C2, and C5) contained the THC/VEA mixture prepared by the laboratory. Two cartridges (Illegal and Authentic) contained unknown mixtures which included Δ^9^-tetrahydrocannabinol. Each cartridge was vaped for three puffs at 3.0 V, then the set voltage was increased by 0.5 V. These steps were repeated until the heating element failed prior to the completion of the three puffs, or until the voltage increase would result in power exceeding the device limit of 30 W. Heating element failure was determined by a failure to produce vapor and a subsequent failure state reading by the device. Temperature was recorded throughout each experiment.

Figure 3 shows the average maximum temperature at each power setting. Power (P in watts) was calculated from the applied voltage (V in volts) and the resistance (R in ohms) of each cartridge by combining Ohm’s and Joule’s Law.

$$P=\frac{V^{2}}{R}$$

As anticipated, temperature increased with power in all cases. In all five cartridges ample vapor was produced with each puff at applied voltages between 3.0 and 5.0 V, corresponding to calculated powers ranging from 4.5 to 16.8 W, with temperatures ranges of 217–300°C at 3.0 V and 415–476°C at 5.0 V. Four cartridges out of six (C2, C5, Illegal, Authentic) produced vapor at 5.5 V, at calculated powers from 15.1 to 18.9 W and a temperature range of 454–510°C. Only three cartridges (C2, Illegal, Authentic) were successfully vaped at 6.0 V, at powers of 18–22.5 W and temperatures of 548–632°C. No vaping was done above 6.0 V due to the power limit of the battery. The temperature response to power of these five cartridges was fit to a linear model with a coefficient of determination (R^2^) of 0.926.

The effects of sustained vaping at elevated power levels on the composition of the THC/VEA mixture, were evaluated by collecting vapor condensate from six cartridges after extended vaping runs (25+ puffs) for analysis (Table 1). Based on the temperature responses observed in the previous experiment, we selected an approximate power target of 16 W (representing applied voltages of 4.7–5.3 V) for vaping of five of the six cartridges. Previous experience with a cartridge similar to C1 suggested that 16 W was beyond the capacity of that cartridge’s heating coil, therefore a power of 12.7 W (4.5 V applied) was selected for C1.

**TABLE 1:** Average vaped per puff determined by change in mass of the cartridge before and after vaporization/total puffs. %Recovery of vaped material determined from the mass of condensate collected/calculated total weight of THC/VEA mixture consumed. The average temperature excludes the first ten vapes to optimize thermocouple placement.

Cartridge	Applied Voltage (V)	Calculated Power (Watts)	Average Vaped per puff (mg)	% Recovery of Vaped Material	Average Temperature ± SD and Range °C	VEA %Recovery ± SD	THC %Recovery ± SD
C1	4.5	12.7	12.1	75.3	439 ± 22 (387–466)	78.9 ± 1.6	69.1 ± 1.5
C2	4.7	15.8	15.1	83.3	441 ± 8.0 (429–461)	100 ± 2.7	88.0 ± 1.3
C3	5.0	15.6	10.7	73.2	456 ± 37 (378–569)	82.0 ± 0.7	62.4 ± 1.6
C4	4.7	15.8	9.04	69.3	445 ± 17 (421–477)	91.4 ± 2.2	73.2 ± 0.1
C5	5.3	15.6	9.05	55.5	503 ± 23 (469–567)	71.5 ± 0.1	56.4 ± 0.2
C6	4.9	16.0	13.9	87.6	448 ± 22 (375–493)	101 ± 3.2	85.0 ± 0.7

Vapor condensate was collected with yields ranging from 55.5 to 87.6% of the calculated total weight of THC/VEA mixture consumed. The likely determinant of recovery yields are the proportions of vapor that escaped from below the cartridge during the initial 3 s of battery operation prior to activation of the pump. In some instances, the amount of vapor seen escaping from the bottom cartridge inlets was significant especially at higher temperatures. The recovery reported here is significantly higher than previously reported for nicotine based products. (Olmedo et al., 2016). Differences in the calculated material consumed per puff, which ranged from 9.04 to 15.1 mg, are presumed to be due in part to variations in cartridge geometry.

The reported temperature in Table 1 is the average maximum temperature recorded per puff, with the first ten measurements excluded from the average because of the time it takes to find the center and hottest location in the coil. Excluding the first ten measurements of each run results in a measured average maximum temperature range of 439 ± 22 (C1) to 503 ± 23°C (C5), with individual minimum and maximum measurements of 375 and 569°C, respectively. Excluding C1, the average maximum temperature (excluding the first 10 measurements) of the five cartridges vaped at a target power of approximately 16 W was 459°C. The temperatures measured in this work are higher than those commonly reported for e-cigarette products intended for use with nicotine based e-liquids, but similar to the temperature reported for a stainless steel coil saturated with VEA. (Geiss et al., 2016; Mikheev et al., 2020).

In order to determine the effect these temperatures have on the major components of cartridges associated with EVALI the concentration of both Δ^9^-THC and VEA were compared to the initial unvaped material. Four of the six cartridges showed reduced recovery for VEA in the range of 71.5–91.4%. Two cartridges exhibited no change though all were vaped at similar temperatures. To further examine this difference two additional sample mixtures were vaped in cartridge C2. This resulted in VEA recoveries of 103 and 109% of the initial VEA concentration. Differences in the recovery of VEA between cartridges is believed to be due in part to variations in cartridge geometry and air flow within the cartridges. All six cartridges showed reduced recoveries for Δ^9^-THC with a range of 56.4–88.0% recovery compared to the unvaped portion. In general, the trend for recovery of Δ^9^-THC correspond to that of VEA for all vaped cartridges. For quality control purposes the material collected from one cartridge, C6 was fortified with Δ^9^-THC and VEA resulting in a 100 and 90% recovery, respectively.

### Analysis of Collected Aerosol

**FIGURE 4:**
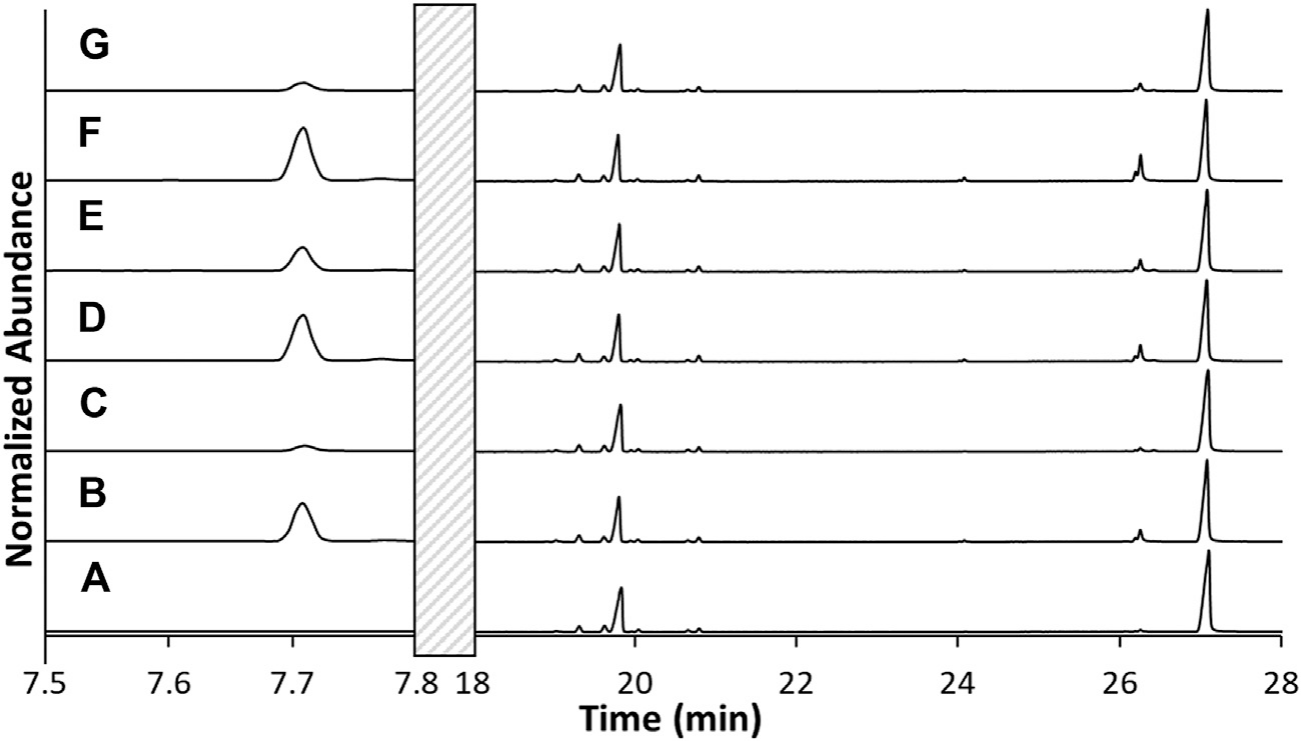
TIC of the unvaped THC and VEA-containing E-liquid **(A)** compared to TICs of the post-vaped E-liquids from cartridges C1-C6 **(B–G)**, respectively. No peaks from 7.8 to 18 min.

GC/MS analysis was performed on the unvaped sample (blank) and the material from the six vaped cartridges (C1, C2, C3, C4, C5, and C6) to determine if additional compounds were produced during vaping. As shown in Figure 4 and Supplementary Figure S2, all samples yielded peaks at 19.8 and 27.1 min that exhibited mass spectra corresponding to Δ^9^-tetrahydrocannabinol (Δ^9^-THC) and vitamin E acetate (VEA), respectively. A comparative analysis of the unvaped sample and the six vapor condensates indicated significant differences in the 5–8 min range. Based on a GC/MS library match, one of the peaks observed was determined to be duroquinone (DQ). Two additional peaks in this range were suspected to be 1-pristene and durohydroquinone monoacetate (DHQMA) based on mass spectral interpretation of the ions that were exhibited in these peaks. This range was further examined using GC/FT-IR/MS to verify the composition of these additional compounds. Additionally, a peak in the derivatized data was observed that supports the presence of DHQMA.

**FIGURE 5:**
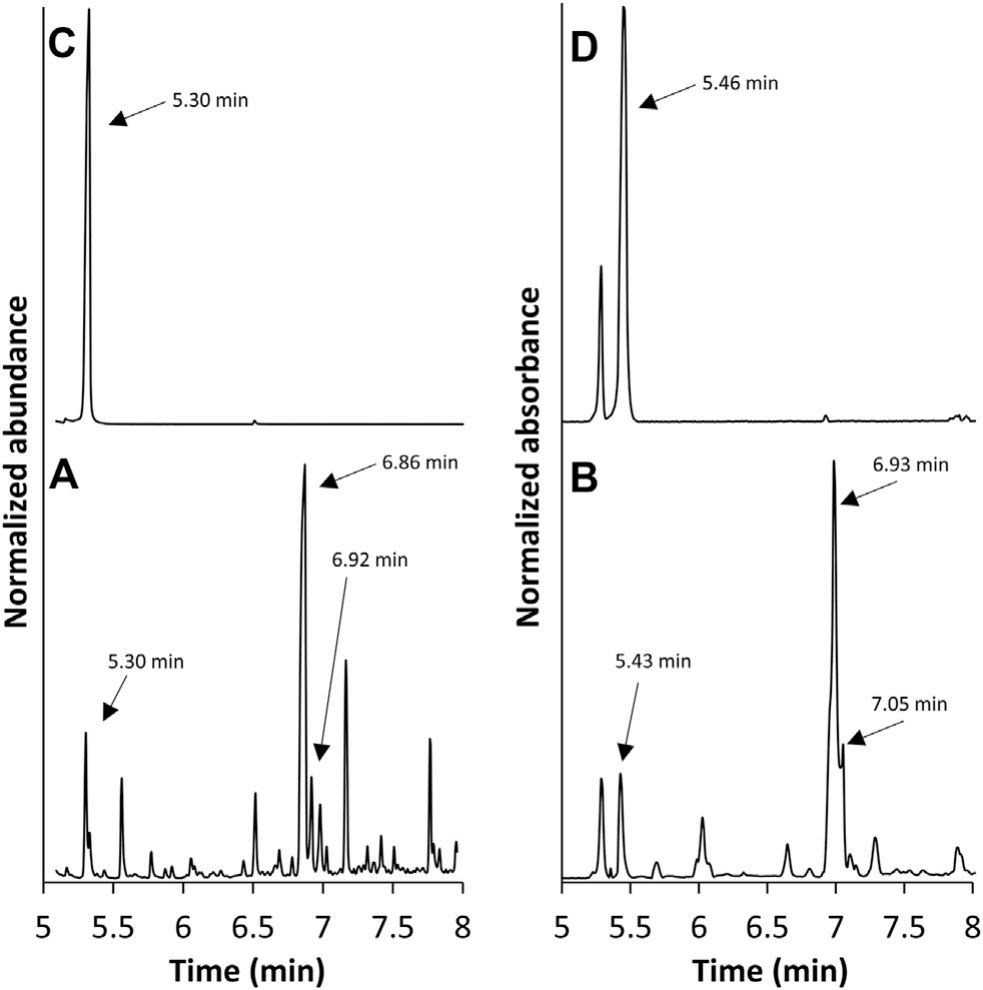
GC/FT-IR/MS TIC **(A)** and AC **(B)** of the post-vape E-liquid from cartridge C5 along with the TIC **(C)** and AC **(D)** of a duroquinone standard.

**FIGURE 6:**
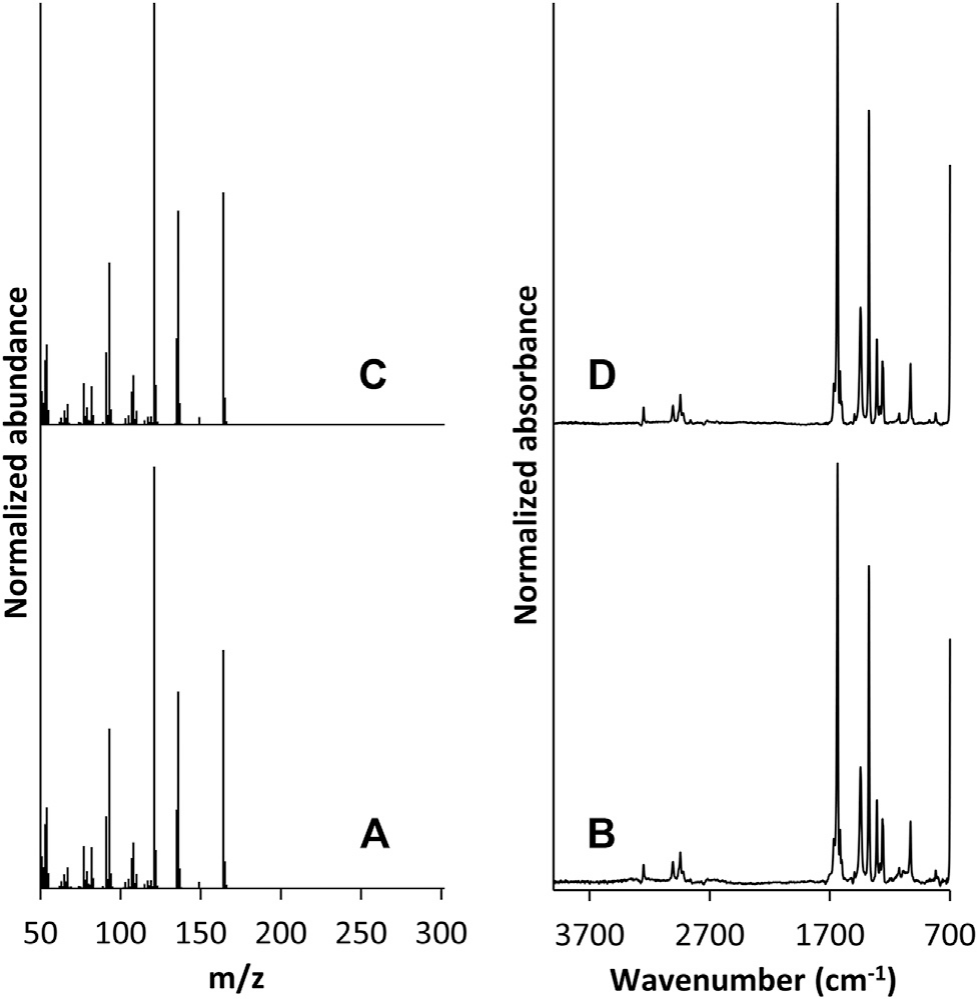
GC/FT-IR/MS mass spectrum of the peak at 5.30 min in the suspect chromatogram from cartridge C5 **(A)** and corresponding IR spectrum **(B)**. Mass spectrum of the peak at 5.30 min in the duroquinone chromatogram **(C)** and corresponding IR spectrum **(D)**.

GC/FT-IR/MS analysis was performed on the collected material from cartridge C5. The total ion chromatogram (TIC) and absorbance chromatogram (AC) of C5 in the 5–8 min region are shown in Figure 5. The C5 extract yielded multiple peaks that were not present in the blank, unvaped sample; the TIC peaks at 5.30, 6.86, and 6.92 min correlate with the AC peaks at 5.43, 6.93, and 7.05 min, respectively. The retention times of the TIC peak at 5.30 min and corresponding AC peak at 5.43 min were consistent with those of the duroquinone standard. Mass and IR spectra corresponding to these peaks in the C5 extract are shown in Figures 6A,B, respectively, and are each consistent with spectra of the duroquinone standard, which are shown in Figures 6C,D. Both compounds exhibited significant ions at *m/z* 164 (molecular ion), 136, 121, 108 and 93 and both compounds exhibited characteristic infrared absorptions at 1,641, 1,448, 1,380, and 1,026 cm^−1^.

The mass spectrum of the peak at 6.86 min in the C5 TIC is shown in Supplementary Figure S3A and the corresponding IR spectrum of the peak at 6.93 min in the C5 AC is shown in Supplementary Figure S3B. The mass spectrum exhibited significant ions at *m/z* 266, 196, 140, 126, and 111; this pattern of loss indicates that the compound is likely a hydrocarbon chain. The IR spectrum indicates a CH_2_ chain-containing hydrocarbon that contains branching CH_3_ groups and a terminal C=C group (vinylidene) in a cis configuration. Specific peak assignments are provided in Supplementary Table S1. Both IR and mass spectra support the structure of 1-pristene.

The mass spectrum of the peak at 6.92 min in the C5 TIC is shown in Supplementary Figure S3C and the corresponding IR spectrum of the peak at 7.05 min in the C5 AC is shown Supplementary Figure S3D. The TIC peak at 6.92 min exhibited significant ions at m/z 208, 166, 151, and 43; the ion at m/z 166 represents a loss of 42 Da that could be due to a loss of an acetyl moiety and the addition of a hydrogen, an ion at m/z 43 indicates the presence of the acetyl moiety. The ion at m/z 151 represents a loss of 15 Da from the ion at m/z 166, which could be due to loss of a methyl group.19,21 The IR spectrum from the AC peak at 7.05 min exhibited many of the same absorptions observed in the IR spectrum from the AC peak at 6.93 min. However, since the AC peak at 7.05 min is not baseline resolved from the peak at 6.93 min, it is unknown if these shared absorptions can be assigned to the peak at 7.05 min or if they are simply carryover from the peak at 6.93 min. Nevertheless, the IR spectrum of the AC peak at 7.05 min clearly exhibited additional absorptions at 3470, 1740, 1,229, and 1,069 cm^−1^ that were not observed in the IR spectrum of the AC peak at 6.93 min. The peak at 3470 cm^−1^ may be assigned to OH stretching vibration, the peak at 1740 cm^−1^ may be due to an acetate ester C=O stretching vibration and the peak at 1,229 cm^−1^ may be due to an acetate ester C-C-O stretching vibration. Both IR and mass spectra support the structure of durohydroquinone monoacetate (DHQMA).

### LC-APCI-HRMS/MS Analysis

The acquired data were interrogated for the presence of the predicted VEA degradants (Wu and O’Shea, 2020; Mikheev et al., 2020) ATMMC, DHQMA, and DQ; the other expected VEA degradants, 1-pristene, and ketene, are not detectable by this analysis. Features consistent with ATMMC, DHQMA, and DQ based on accurate mass measurement were observed in each of the vaped condensates, but not observed in the unvaped sample (see Supplementary Figures S4–S9). DQ was further identified by comparison to a reference standard (Supplementary Figures S8, S9) and, as described above, a compound consistent with DHMQA was also detected by GC/MS analysis of both the derivatized and non-derivatized vaped condensates, as well as by GC/FT-IR/MS analysis; no compounds consistent with ATMMC were observed by GC/MS or GC/FT-IR/MS, perhaps due to lower abundance, which would be consistent with its proposed role as a reactive degradation intermediary. (Mikheev et al., 2020).

**FIGURE 7:**
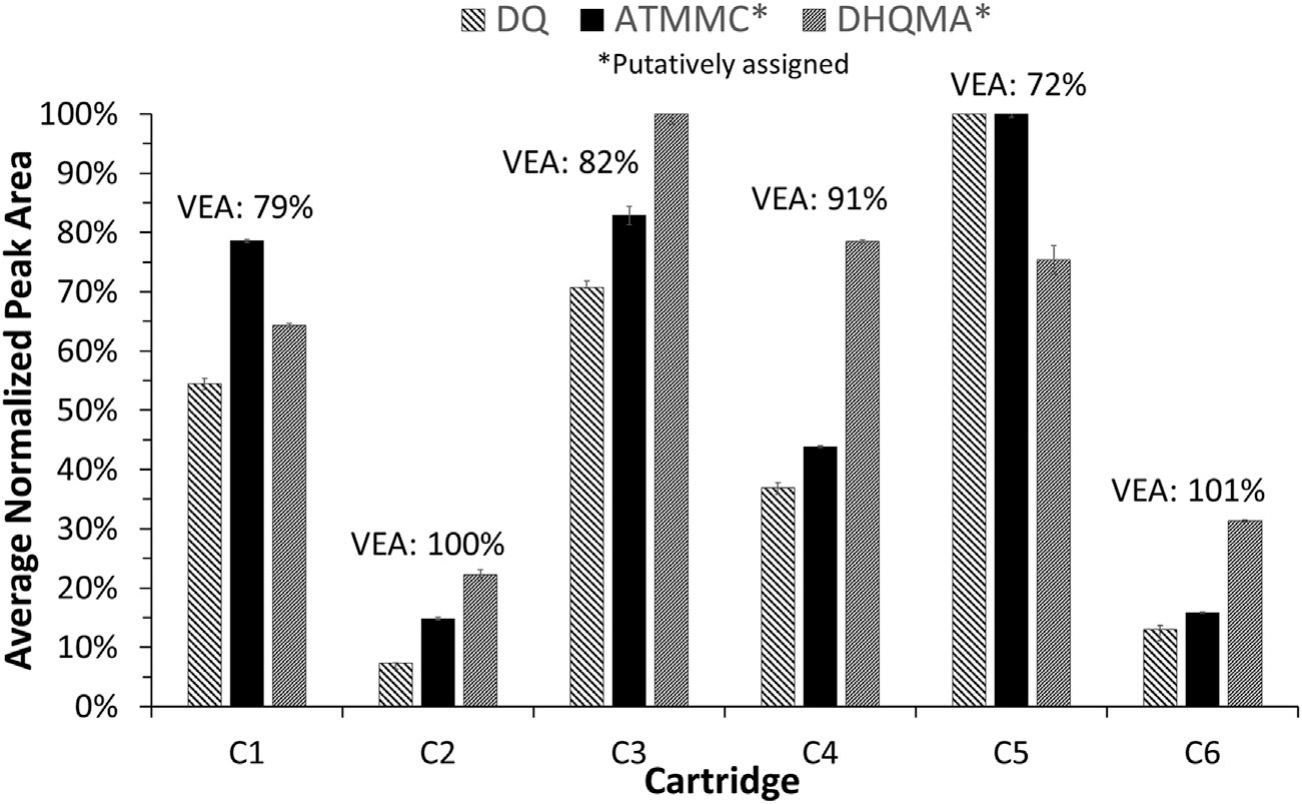
Peak areas, normalized to the cartridge with the largest peak area for each compound, measured in the vaped samples for duroquinone (using the LC-MS EIC of *m/z* 165.0910), putatively assigned ATMMC (using the LC-MS EIC of *m/z* 207.1016), and putatively assigned DHQMA (using the LC-MS EIC of *m/z* 149.0962), reported as the average of three injections. Error bars represent two standard deviations. The measured recovery of VEA for each cartridge is shown above each cluster in the graph for reference.

From their respective EICs, the peak areas were measured for these three compounds and compared to the recoveries of VEA measured for each cartridge. If these compounds are formed from the degradation of VEA, it is expected that larger peak areas would be observed for those samples that exhibited the lowest VEA recoveries, and this is indeed the case as shown in Figure 7. As shown in Table 1, cartridge C5 yielded the lowest VEA recovery, 71.5%, and the largest peak areas for DQ, putatively assigned ATMMC, and putatively assigned DHQMA. Similarly, cartridges C1 and C3 yielded the next lowest VEA recoveries, approximately 80%, and the next largest peak areas for DQ and putatively assigned ATMMC. Cartridges C2 and C6 exhibited the highest VEA recoveries, approximately 100%, and the smallest peak areas for DQ and putatively assigned ATMMC. The peak areas for putatively assigned DHQMA exhibited a similar pattern of relative abundances, with a couple of exceptions: cartridges C3 and C4 yielded a higher abundance than expected, and cartridge C5 a lower abundance than expected, relative to the other cartridges based on the VEA recoveries.

Although the presence of ketene could not be detected directly, the proposed reaction schemes that explain its formation indicate that one molecule of ketene is formed for each molecule of DQ that is formed. (Wu and O’Shea, 2020; Mikheev et al., 2020). Therefore, measurement of the amount of DQ can be used as an indirect measurement of the amount of ketene generated. Standard additions were used to measure the level of DQ in the vaped liquid collected from cartridge C1. The EIC of the product ion *m/z* 107.0495 was used to obtain the peak areas since it had the lowest background in the region of the DQ peak (Supplementary Figure S10). The calibration curve obtained is shown in Supplementary Figure S11, in which the dots represent the average peak areas, measured from triplicate injections. The calculated concentration of DQ in the analyzed solution of the vaped condensate collected from cartridge C1 was 8.74 ± 0.14 μg/ml, which corresponds to 4.16 ± 0.07 μg DQ per mg of vaped condensate, or 0.416 ± 0.007% by mass (the uncertainties represent two standard deviations). This results in a VEA to DQ conversion rate of approximately 1%, which is significantly less than that shown using cartridges with lower resistance and higher power. (Wu and O’Shea, 2020). Using the information from Table 1 that 12.1 mg of sample were vaped per puff for cartridge C1, the measured DQ level corresponds to an average of 50.4 μg DQ generated per puff and, by extension, an estimated maximum of 12.9 μg ketene generated per puff.

The qualitative LC-APCI-HRMS/MS data were also evaluated for the presence of additional compounds that were present either only in the vaped condensates or at visibly higher levels in the vaped condensates than the unvaped liquid. The chromatograms of the vaped condensates contained many features that were not observed in the unvaped liquid. The major features were tentatively assigned as isomerization or degradation products of Δ^9^-THC and/or other cannabinoids present in the sample based on molecular formulae generated from HRMS data that corresponded to compounds previously observed in thermal decomposition studies of cannabinoids. (Mechoulam, 1970; Küppers et al., 1975; Salemink, 1976; Spronck et al., 1978; Tjeerdema, 1987). An in-depth investigation of the many features present in the LC-MS and derivatized GC/MS data is beyond the scope of this study and will be the focus of future work.

## Conclusion

In this study we establish that vaping using a commercially available device and ceramic coil cartridges can produce coil temperatures sufficient for the thermal decomposition of VEA in THC/VEA mixtures. The initial VEA concentration in the THC/VEA mixture was reduced following vaporization in four of the six cartridges, though all cartridges achieved similar maximum temperatures under the experimental conditions in this work. The initial concentration of THC in the THC/VEA mixture was reduced following vaporization in all six cartridges. These results were generated using elevated power settings, and the congruence of this method with typical vaping practices among consumers is presently unexamined. Additionally, we identified compounds consistent with the production of ketene gas by proposed pathways for VEA decomposition, (Wu and O’Shea, 2020) including detection of a substance that, based on exact mass measurement, was consistent with the previously undetected quinone methide ATMMC. Although ketene itself was not detected by the methods employed in this study, detection of the previously identified VEA degradants (Jiang et al., 2020; Mikheev et al., 2020; Wu and O’Shea, 2020)in the vaped condensates collected from the THC/VEA mixtures indicates that, at least under some conditions that consumers may encounter, pulmonary toxins may be produced from vaping of mixtures containing VEA in ceramic coil cartridges.

## Data Availability

The original contributions presented in the study are included in the article/Supplementary Material, further inquiries can be directed to the corresponding author.
