# Supplementary material for: Simultaneous Temperature Measurements and Aerosol Collection During Vaping for the Analysis of Δ9-Tetrahydrocannabinol and Vitamin E Acetate Mixtures in Ceramic Coil Style Cartridges
Source: Front Chem. 2021 Aug 9;9:734793. doi: 10.3389/fchem.2021.734793 (PMC8381023; doi:10.3389/fchem.2021.734793)
Supplement: Supplementary file 1 [file DataSheet1.docx]

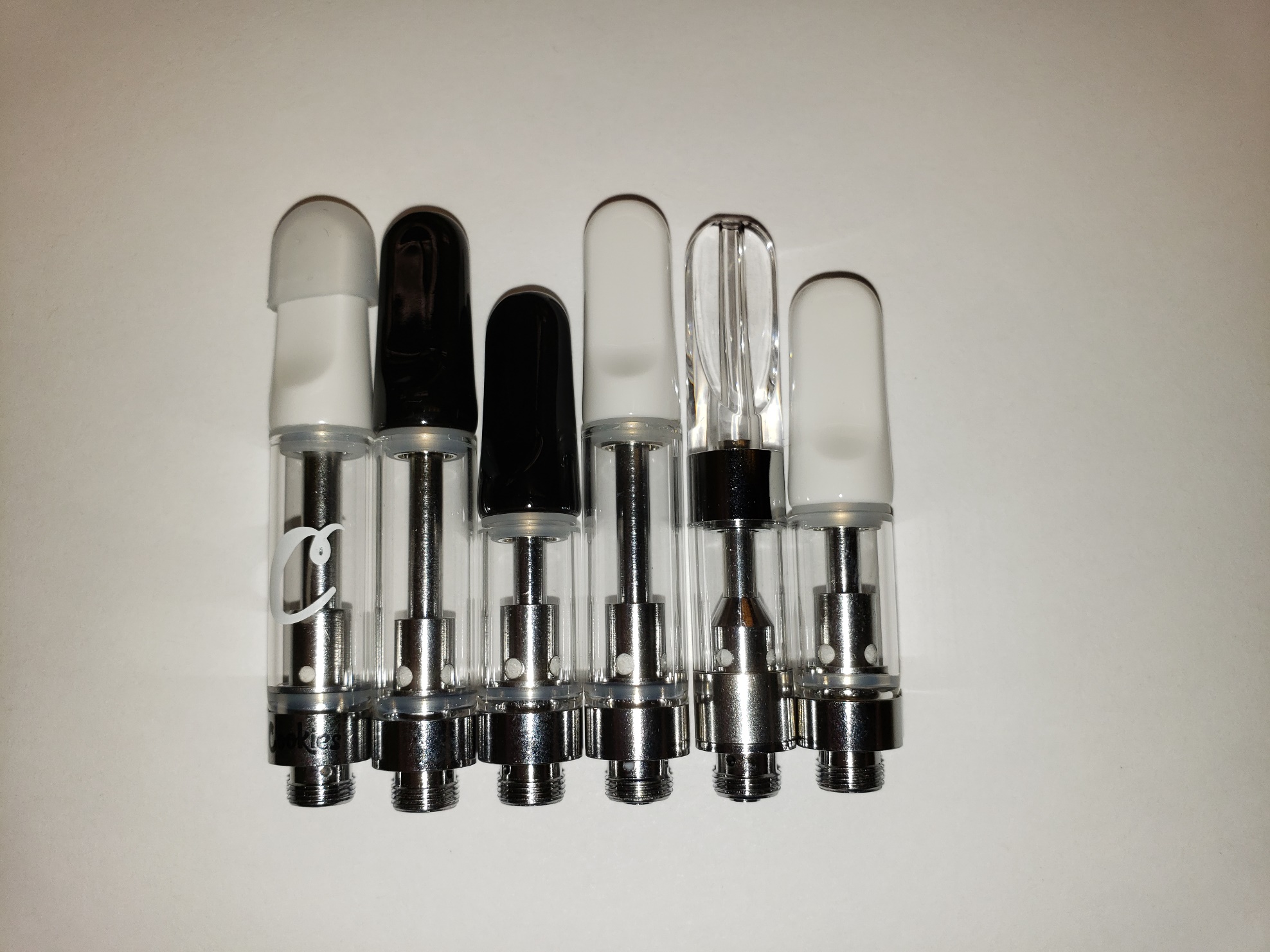


Supplementary Figure 1. Image of empty ceramic coil cartridges used in this work. Identified C1-C6 from left to right. Measured resistances of cartridges C1 through C6 prior to use were 1.6, 1.4, 1.6, 1.4, 1.8, and 1.5 Ω, respectively.


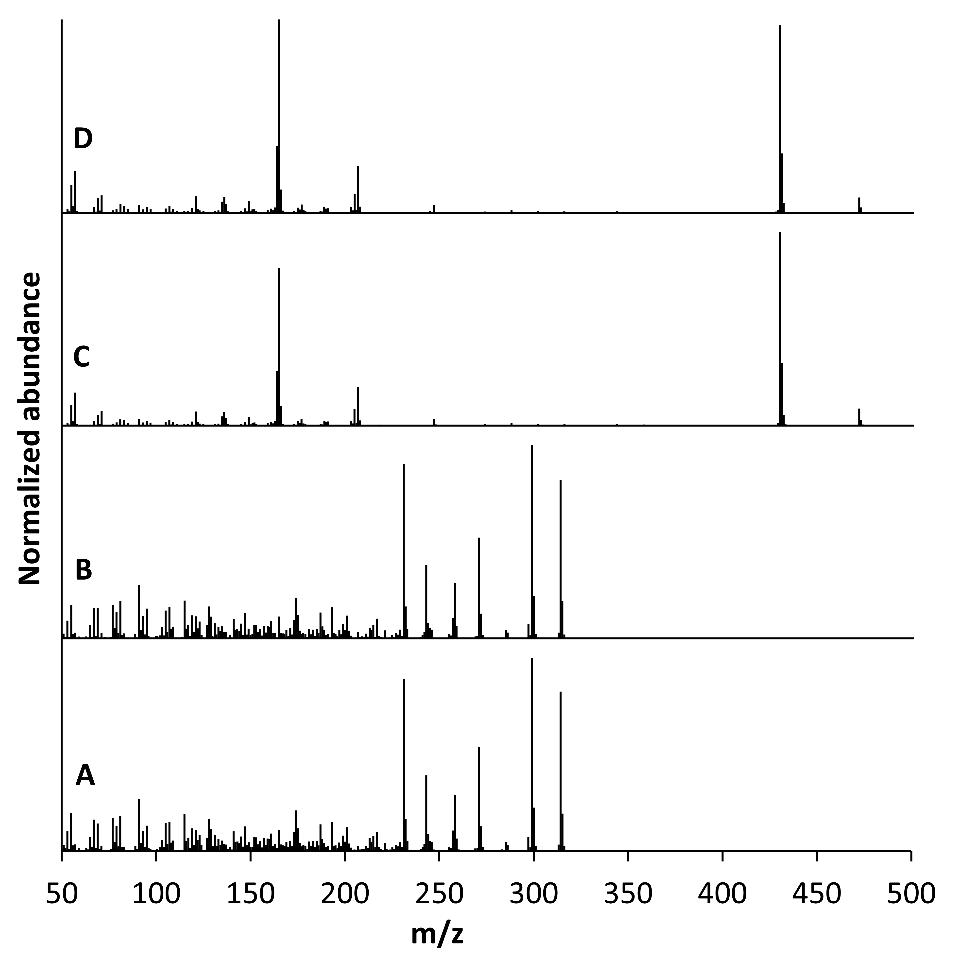


Supplementary Figure 2. Mass spectrum of the peak at 19.79 min in the suspect total ion chromatogram of unvaped material (A), library spectrum of a Δ^9^-THC standard (B), mass spectrum of the peak at 27.07 min in the suspect total ion chromatogram of unvaped material (C) and library spectrum of VEA (D).


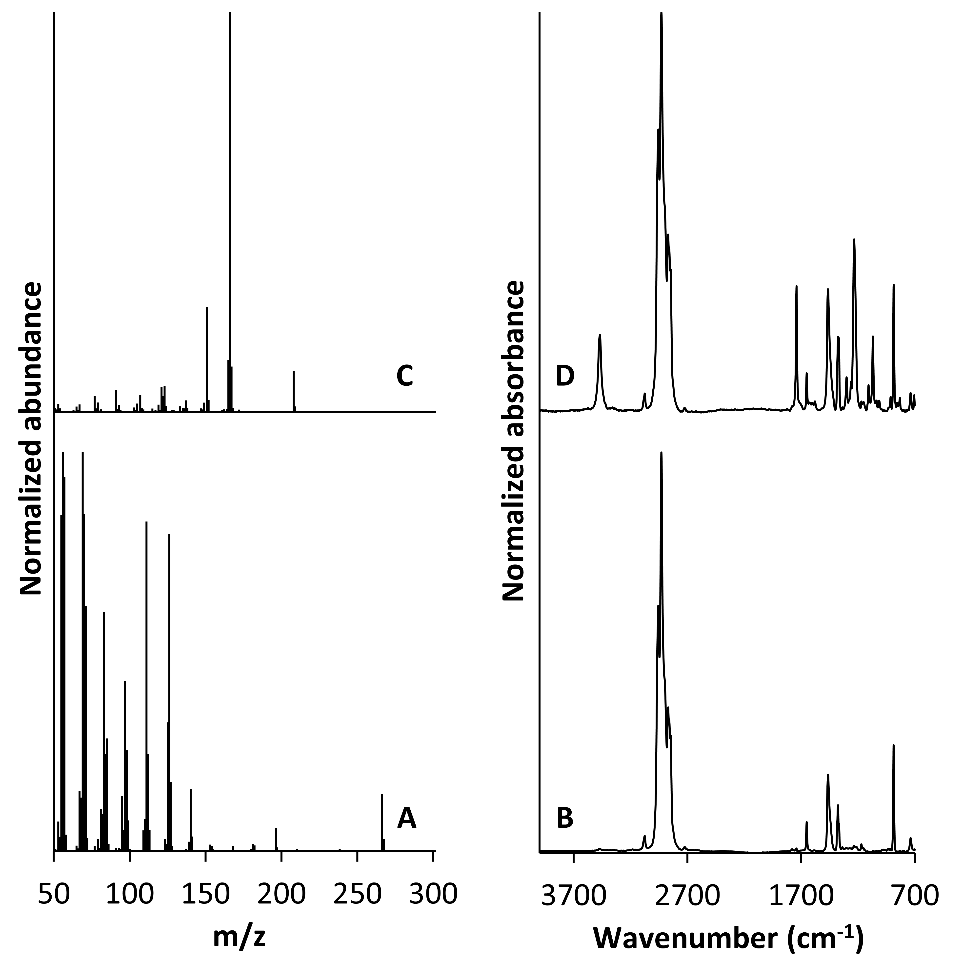


Supplementary Figure 3. GC/FT-IR/MS mass spectra of the TIC peaks at 6.86 and 6.92 min from cartridge C5 (A and C) and corresponding IR spectra of the AC peaks at 6.93 and 7.05 min (B and D), respectively.


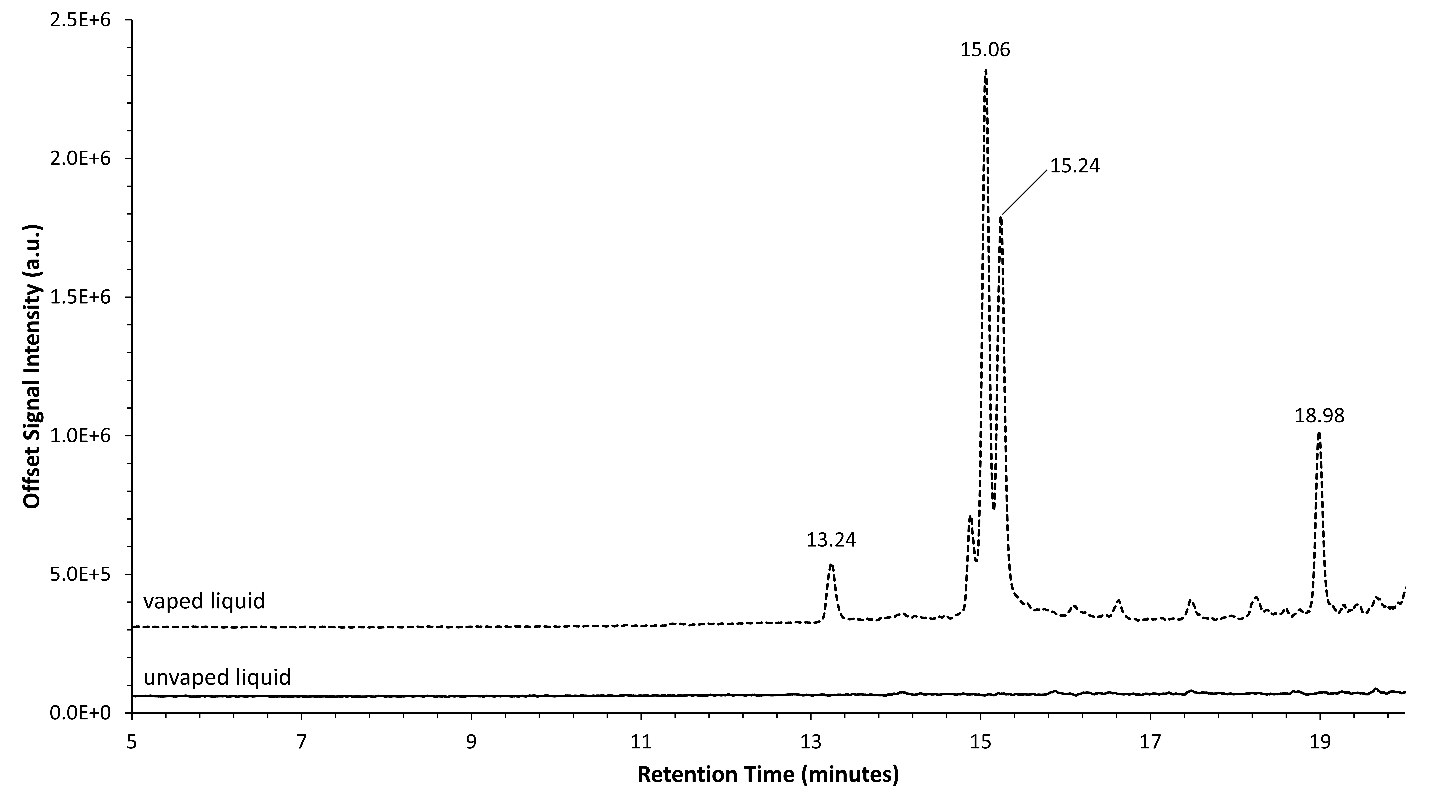


**Supplementary Figure 4.** LC-MS extracted ion chromatograms of *m/z* 207.1016 (±5 ppm). Top: vaped liquid (C5); feature at *t_R_* = 15.06 minutes is putatively assigned as 4-acetoxy-2,3,5-trimethyl-6-methylene-2,4-cyclohexadienone (ATMMC). Bottom: unvaped liquid (B).


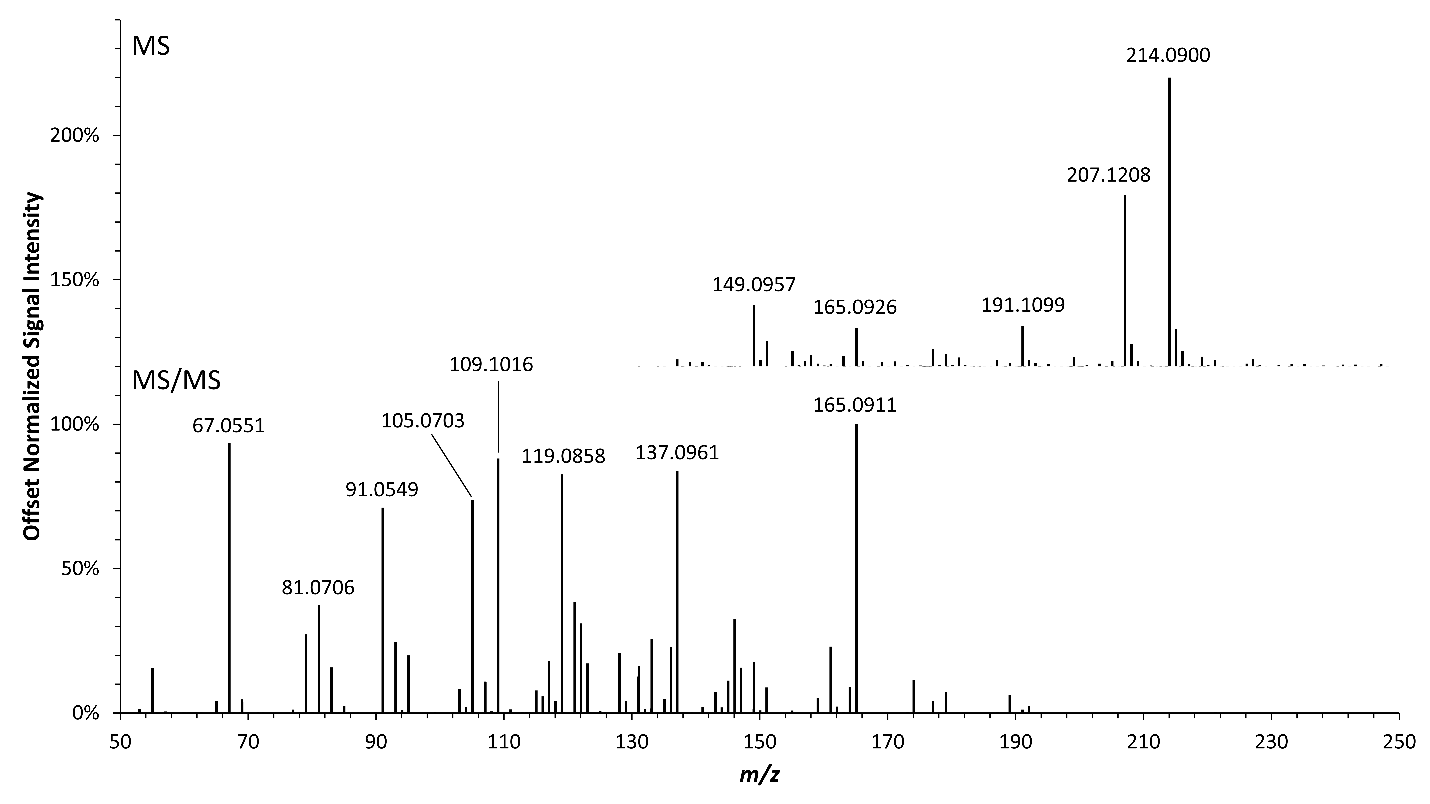


**Supplementary Figure 5.** LC-MS spectra from vaped liquid (C5) feature at *t_R_* = 15.06 minutes putatively assigned as 4-acetoxy-2,3,5-trimethyl-6-methylene-2,4-cyclohexadienone (ATMMC). Top: full scan mass spectrum. Note that *m/z* 214.0900 is also present in the unvaped liquid at this retention time. The other labeled ions are present only in the vaped liquid at this retention time. Bottom: full scan MS/MS spectrum with precursor *m/z* 207.1 ± 0.5.


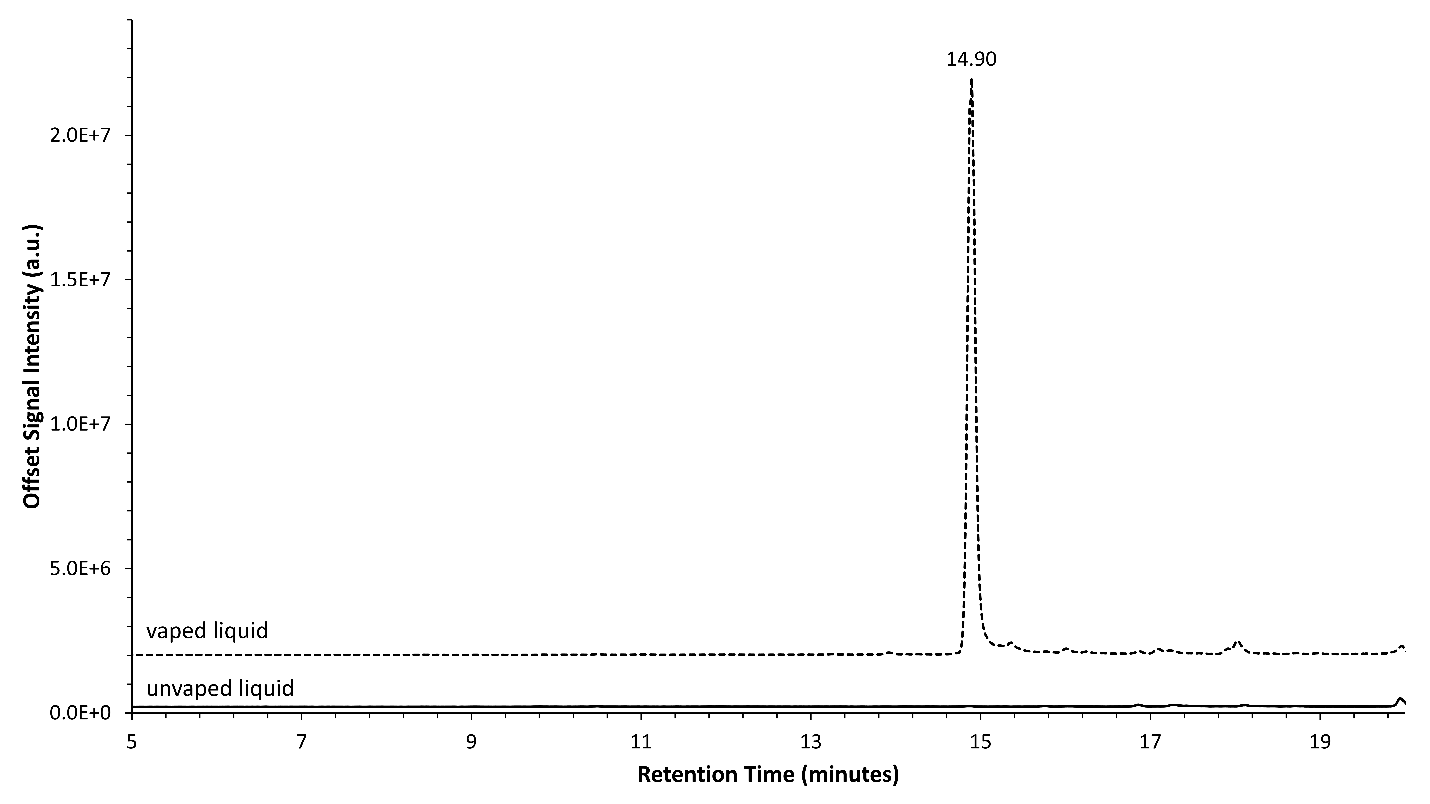


**Supplementary Figure 6.** LC-MS extracted ion chromatograms of *m/z* 149.0962 (±5 ppm; assigned as an in-source fragment of *m/z* 209.1209). Top: vaped liquid (C5); feature at *t_R_* = 14.90 minutes is putatively assigned as durohydroquinone monoacetate (DHQMA). Bottom: unvaped liquid (B).


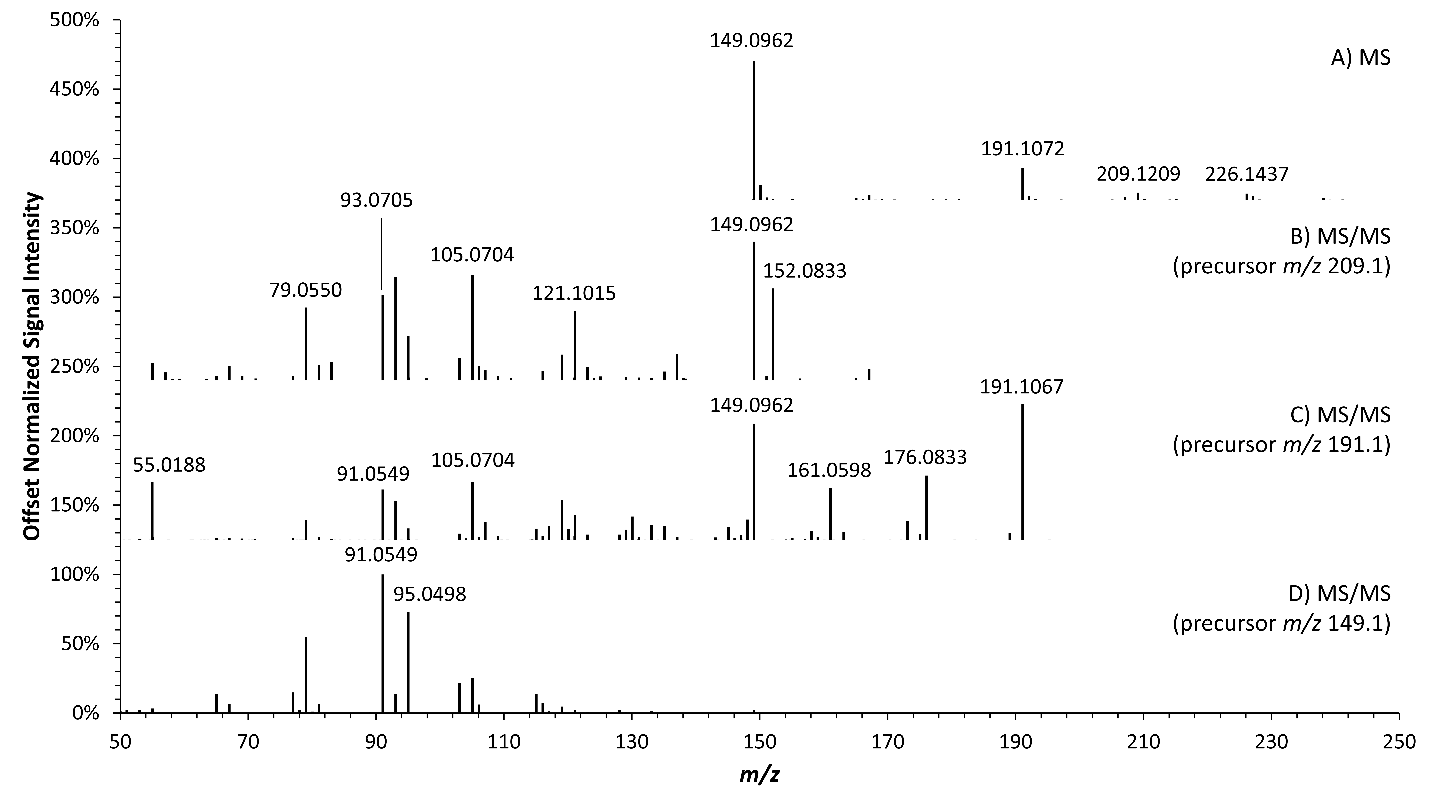


**Supplementary Figure 7.** LC-MS spectra from vaped liquid (C5) feature at *t_R_* = 14.90 minutes putatively assigned as durohydroquinone monoacetate (DHQMA). A) Full scan mass spectrum. Ions assigned as follows: 226.1437, [M+NH_4_]^+^; 209.1209, [M+H]^+^; 191.1072, [M+H-H_2_O]^+^; 149.0962, [M+H-C_2_H_4_O_2_]^+^. B) Full scan MS/MS spectrum with precursor *m/z* 209.1 ± 0.5. C) Full scan MS/MS spectrum with precursor *m/z* 191.1 ± 0.5. D) Full scan MS/MS spectrum with precursor *m/z* 149.1 ± 0.5.


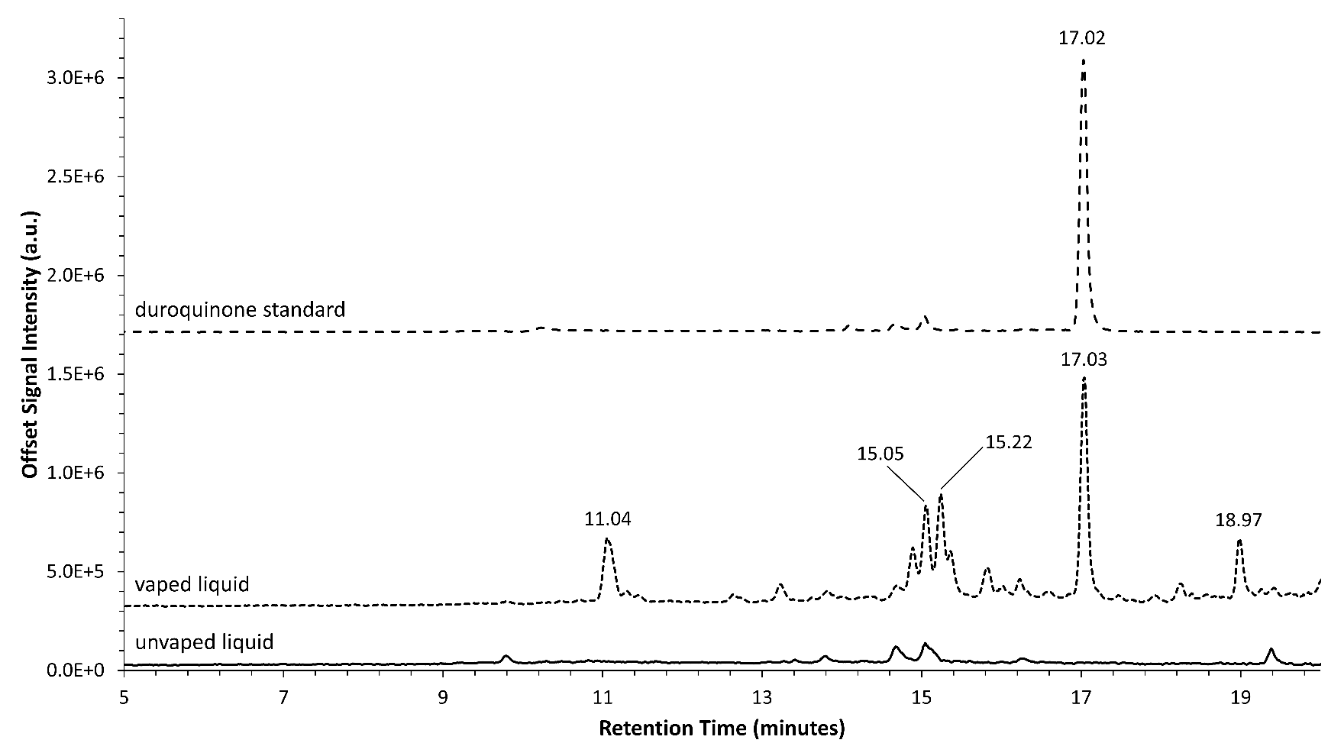


**Supplementary Figure 8.** LC-MS extracted ion chromatograms, *m/z* 165.0910 (±5 ppm). Top: ~2 ppm duroquinone standard. Middle: vaped liquid (C5); feature at *t_R_* = 17.03 minutes is identified as duroquinone (DQ). Bottom: unvaped liquid (B).


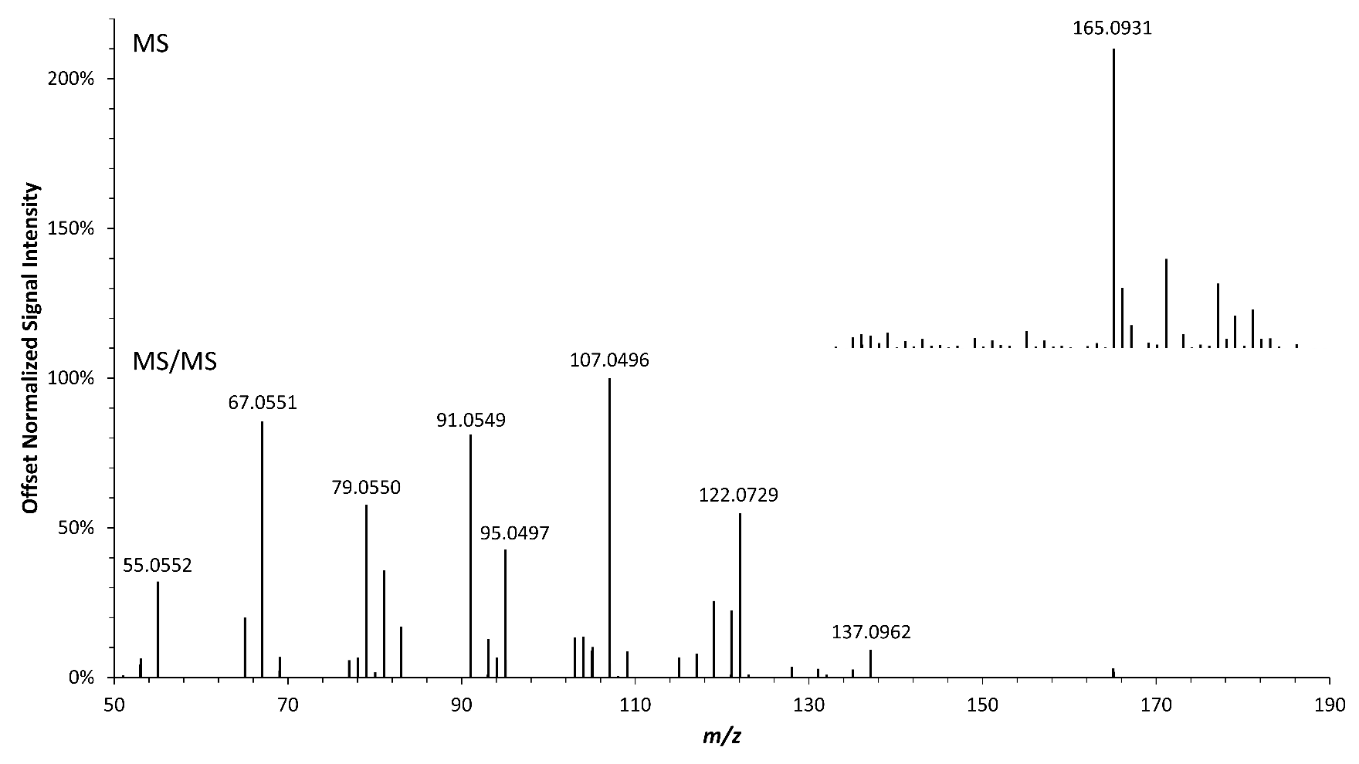


**Supplementary Figure 9.** LC-MS spectra from vaped liquid (C5) feature at *t_R_* = 17.03 minutes identified as duroquinone. Top: full scan mass spectrum. Bottom: full scan MS/MS spectrum with precursor *m/z* 165.1 ± 0.5, which is consistent with the MS/MS spectrum of duroquinone reference standard obtained at this retention time.


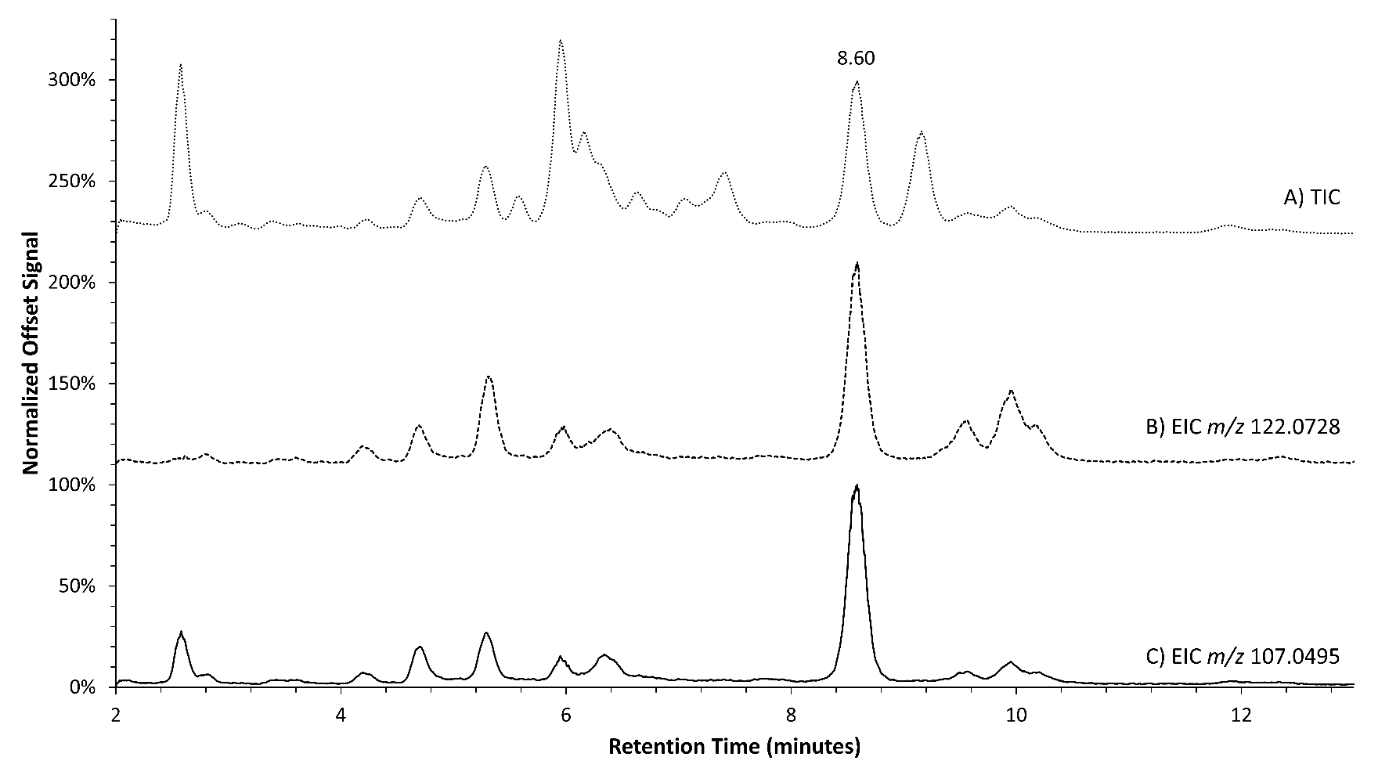


**Supplementary Figure 10.** LC-MS chromatograms of vaped liquid from cartridge C1 obtained by the method used for quantitative analysis. The peak at *t_R_* = 8.60 minutes corresponds to duroquinone. A) Total ion chromatogram. B) Extracted ion chromatogram of product ion *m/z* 122.0728 (±5 ppm). C) Extracted ion chromatogram of product ion *m/z* 107.0495 (±5 ppm).


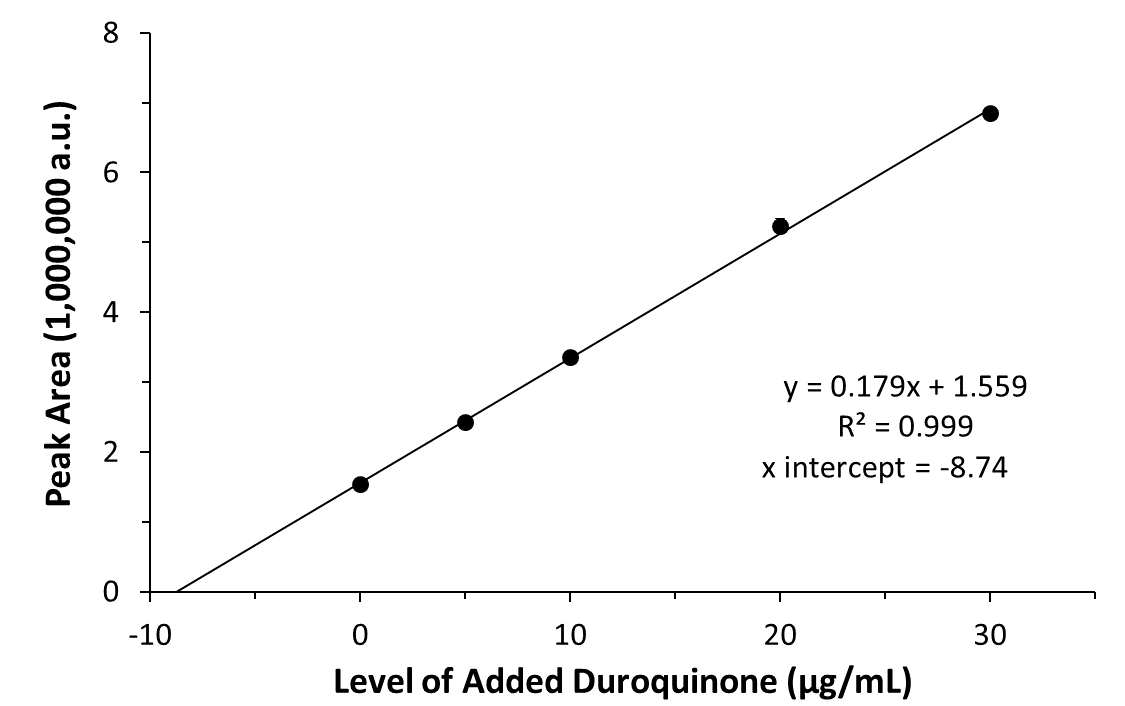


Supplementary Figure 11*.* Standard additions calibration curve generated by plotting peak areas obtained using extracted ion chromatograms of the product ion *m/z* 107.0495 (±5 ppm) vs the level of added duroquinone. Error bars representing two standard deviations are smaller than the data markers.

Supplementary Table 1 Band assignments for the infrared spectrum corresponding to the AC peak at 6.93 min.

| Peak (cm^-1^) | Assignment |
| --- | --- |
| 3075 | C=C-H stretching |
| 2955 | CH_3_ anti-symmetric stretching |
| 2928 | CH_2_ anti-symmetric stretching |
| 2869 | CH_3_ symmetric stretching |
| 2847 | CH_2_ symmetric stretching |
| 1651 | C=C stretching in a cis configuration |
| 1462 | CH_2_ scissoring |
| 1385(shoulder)/ 1375/1366 split peak | CH_3_ umbrella deformation characteristic of branching |
| 886 | Out of plane C-H bending characteristic of a vinylidene moiety |
| 736 | CH_2_ in-phase rocking |
